# Boosting the Efficiency of Photoelectrolysis by the Addition of Non-Noble Plasmonic Metals: Al & Cu

**DOI:** 10.3390/nano9010001

**Published:** 2018-12-20

**Authors:** Qianfan Jiang, Chengyu Ji, D. Jason Riley, Fang Xie

**Affiliations:** Department of Materials and London Centre for Nanotechnology, Imperial College London, London SW7 2AZ, UK; q.jiang16@imperial.ac.uk (Q.J.); c.ji18@imperial.ac.uk (C.J.); jason.riley@imperial.ac.uk (D.J.R.)

**Keywords:** photoelectrolysis, plasmonics, aluminum, copper

## Abstract

Solar water splitting by semiconductor based photoanodes and photocathodes is one of the most promising strategies to convert solar energy to chemical energy to meet the high demand for energy consumption in modern society. However, the state-of-the-art efficiency is too low to fulfill the demand. To overcome this challenge and thus enable the industrial realization of a solar water splitting device, different approaches have been taken to enhance the overall device efficiency, one of which is the incorporation of plasmonic nanostructures. Photoanodes and photocathodes coupled to the optimized plasmonic nanostructures, matching the absorption wavelength of the semiconductors, can exhibit a significantly increased efficiency. So far, gold and silver have been extensively explored to plasmonically enhance water splitting efficiency, with disadvantages of high cost and low enhancement. Instead, non-noble plasmonic metals such as aluminum and copper, are earth-abundant and low cost. In this article, we review their potentials in photoelectrolysis, towards scalable applications.

## 1. Introduction

With the increasing demand for energy, reliable and low-cost systems that can harvest solar energy with high efficiency have been a goal for researchers working across many distinct fields [1]. Solar electrolysis with semiconductor-based photoanodes and photocathodes is one of the promising strategies for solar fuels, where water splits into hydrogen and oxygen, which could be stored and consumed on demand. Solar water splitting, converting solar energy to chemical energy (formation of hydrogen) by semiconductors, is a clean and eco-friendly energy strategy with expanding applications [2]. The performance of water splitting devices is determined by the bandgap, carrier separation distance and stability of the semiconductor. To date, a perfect semiconductor has not been found to match all the requirements for an industrial level water splitting device [3]. To overcome this problem and thus enable the industrial application of a solar water splitting device, different approaches have been taken to enhance the overall device efficiency, one of which is utilizing plasmonic nanostructures in water splitting systems. Plasmonic materials have been studied and used in many fields including biological sensing [4], chemical sensing [5], photocatalysis [6] and metal-enhanced fluorescence [7,8]. Researchers have revealed that plasmonic nanostructures can also enhance water splitting by semiconductors, with various pathways to enhancement being proposed. Photoanodes and photocathodes coupled to optimized plasmonic nanostructures, matching the absorption wavelength of the semiconductors, can potentially exhibit a significantly increased efficiency. 

Gold and silver are the traditional plasmonic materials, they exhibit many fascinating properties and are the most common candidate materials from which to fabricate plasmonic nanostructures [9,10,11,12]. However, the high cost and low earth abundance of these noble metals mean that it is challenging to implement industrial scale light-harvesting devices with gold or silver as the enhancing material. In addition, the wavelength at which gold and silver show plasmonic effects is over a narrow range, disabling their capability of light harvesting across the entire solar spectrum. In light of these drawbacks, other plasmonic materials such as aluminum and copper may be considered. Aluminum and copper are non-noble metals which are earth-abundant, low cost, widely used in industry. These common metals also have wider plasmonic bands that could enable broadband plasmonic enhanced light harvesting. More significantly, aluminum’s plasmonic wavelength is within the UV-Visible range, enabling enhancement at energies greater than the semiconductor band gaps, which in turn induced significant efficiency enhancement due to the mechanism of plasmon-induced resonance energy transfer (PIRET).

## 2. Plasmonic Effects

Metal-dielectric interfaces show extraordinary electromagnetic properties and have raised the interest from researchers in many fields. Mie, G. firstly studied the optical properties of colloidal plasmonic metal nanoparticles. It was found that a different plasmonic property can also be exhibited by metal thin films [13]. Nowadays, the term plasmonic usually refers to two different phenomena, that is localized surface plasmon resonance (LSPR) and surface plasmon polaritons (SPP).

### 2.1. Optical Properties of Plasmonic Metal Nanoparticles

**Figure 1:**
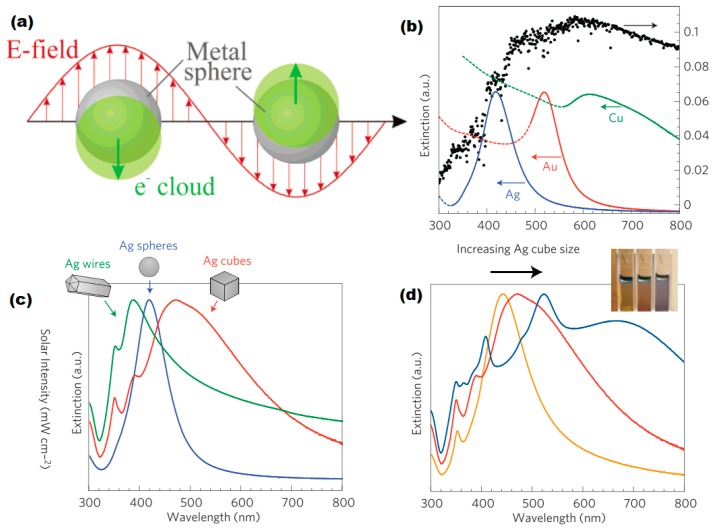
(**a**) Schematic illustration of plasmonic resonant of the free electron cloud around spherical metal particles. Reproduced with permission from [14]. Copyright American Chemical Society, 2003. (**b**) Normalized extinction spectra of Ag, Au and Cu particles, (**c**) Normalized extinction spectra of Ag cube, sphere and wire, (**d**) Size-dependent extinction spectra of Ag nanocubes. Reproduced with permission from [15]. Copyright Springer Nature, 2011.

The particle size related colors of noble metal particles are the most common phenomena from localized plasmonic effects. When the size of the nanoparticles is significantly smaller than the wavelength of the incident light, a resonant electromagnetic behavior is displayed. As illustrated in Figure 1a, the free electrons of plasmonic metal nanoparticles are confined to a small volume and oscillate according to the external electromagnetic fields which lead to polarized charges on the plasmonic surface and generate a local electromagnetic field. At the plasmonic resonance wavelength, the restoring force of the free electrons match the frequency of the plane wave, hence localized plasmonic effects could be observed. The particle dipole plasmonic frequency is highly related to the size, morphology as well as the permittivity of the plasmonic nanoparticles. Figure 1b–d are examples of the correlation of plasmonic extinction spectra and the size and morphology of Ag and/or Au nanoparticles.

By controlling the size and morphology of the plasmonic nanoparticles, the resonance wavelength is optically tunable. However, the achievable wavelength range is limited by the dielectric function of the material itself, therefore choosing an appropriate plasmonic material to match the working frequencies of the device is critically important. Traditional noble plasmonic metals, such as gold and silver, mostly locate at the visible wavelength range, while aluminum and copper can cover a much broader spectral range. The plasmonic range is from 150 nm to 600 nm and from 600 nm to 1100 nm for aluminum and copper, respectively. Therefore, it is possible to design and develop a plasmonic component overlapping with the whole solar energy spectrum using only non-noble metals. In other words, it is possible to harvest the whole solar spectrum from UV to near infrared, which is significant for an efficient photoelectrolysis device.

### 2.2. Surface Plasmon Polaritons at Dielectric/Plasmonic Metal Interfaces

**Figure 2:**
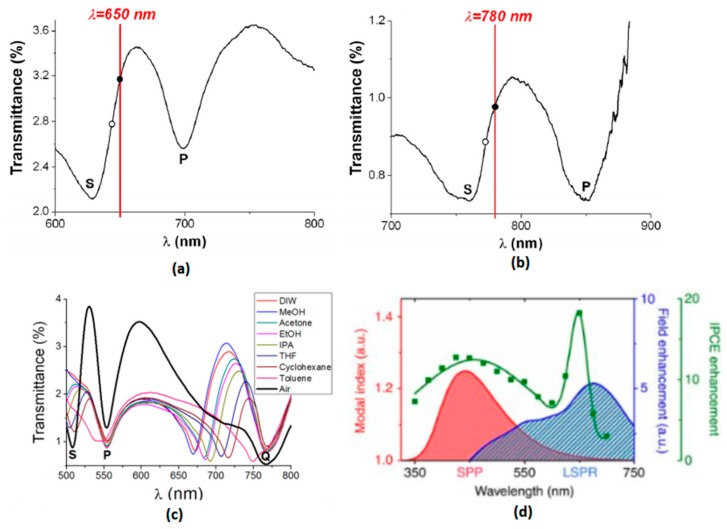
(**a**,**b**) Experimental transmission spectra of 620-nm-period and 750-nm-period aluminum nanohole array indicating their S mode (at the air/metal interface) and P mode (at the glass/metal interface) SPP resonance wavelength, (**c**) Transmission spectra of 500-nm-period aluminum nanohole arrays in different dielectric medium indicating their S mode (at the medium/metal interface) and P, Q mode (at the glass/metal interface) SPP resonance wavelength, [4] (Reprinted from Biosensors under CC-BY license), (**d**) Incident photon to current conversion efficiency (IPCE) enhancement and field enhancement of SPP and LSPR mode from 500-nm-period Au nanohole arrays in hematite photoanodes. Reproduced with permission from [17]. Copyright Springer Nature, 2013.

Surface plasmon polaritons is another instance of plasmonic light-matter interaction. Similar to LSPR, SPP can also create an enhanced electromagnetic field at the metal-dielectric surface. However, in this case, it is the plasmonic metal thin film with periodic nanostructures like nanorods and nanoholes acting as gratings to excite the SPP Bloch waves and thus generate an electromagnetic field across the whole interface continuously [16]. The SPP grating-coupling equation is:

(1) $$\lambda =\;\frac{a}{\sqrt{i^{2}+j^{2}}}\cdot \sqrt{\frac{\varepsilon_{d}\cdot \varepsilon_{m}}{\varepsilon_{d}+\varepsilon_{m}}}$$

where *λ* is the SPP resonance wavelength, *a* is the periodic length of the thin film, εd and εm are permittivities of dielectric and metal respectively and *i*, *j* are integers to determine at which mode is the SPP resonance. From Equation (1) it is clear to see that unlike LSPR resonance, SPP resonance is highly related to the periodic parameters of the thin film, as shown in Figure 2a,b.

For periodic plasmonic thin films, multiple modes of SPP can be excited simultaneously at different frequencies, as demonstrated in Figure 2c, thus enabling the ability of tunable and broadband plasmonic enhancement.

Moreover, the SPP resonance modes usually exist simultaneously with LSPR modes in such periodic plasmonic metal thin films. Due to the same mechanism, localized resonance can also take place in thin film nanostructures especially those with sharp edges and tips. As shown in Figure 2d, in gold nanohole arrays, the SPP mode locates at the spectral range of UV to visible while the LSPR mode covers the visible to NIR regions. Such plasmonic thin films with both LSPR and SPP modes at different wavelengths can enhance the device to a much better extent if the resonance frequencies are optimized to the ideal spectral positions.

## 3. Mechanisms of Plasmonic-Enhanced Photoelectrolysis

**Figure 3:**
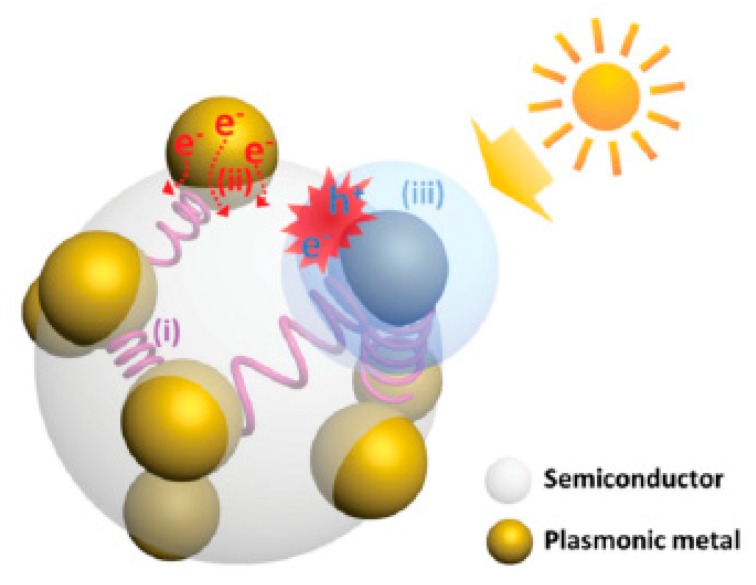
Schematic illustration of three generally accepted mechanisms for plasmonic enhancement: (i) light scattering, (ii) HEI, (iii) PIRET. Reproduced with permission from [18]. Copyright John Wiley and Sons, 2015.

The photoelectrolysis process is essentially a light harvesting process converting the energy from photons to the energy of electron-hole pairs and subsequently to energy in chemical bonding. Many different mechanisms have been proposed to help us understand and clarify the pathways of plasmonic enhancement in the photoelectrolysis process. Among them, three mechanisms have been generally accepted which are near-field excitation enhancement, hot-electron injection (HEI), plasmon induced resonance energy transfer (PIRET) [18], shown in Figure 3. In addition, the photothermal effect has also draw researchers’ attention in recent years [19].

### 3.1. Near–Field Excitation Enhancement

Due to the phenomenon of LSPR and SPP, the electric field enhancements in the proximity of the nanoparticles are expected to result in an increase in the rate of photoexcitation of the semiconductor in semiconductor/plasmonic nanoparticles composite electrodes, given that the rate of charge carrier generation is proportional to |E|^2^ [20]. The key requirement for such pathways is that plasmonic resonance frequency is located at a spectral position above the band gap energy of the semiconductors [21]. This requirement could be used to distinguish near-field excitation enhancements from other pathways of enhancements such as HEI and PIRET discussed below.

### 3.2. Hot Electron Injection (HEI)

**Figure 4:**
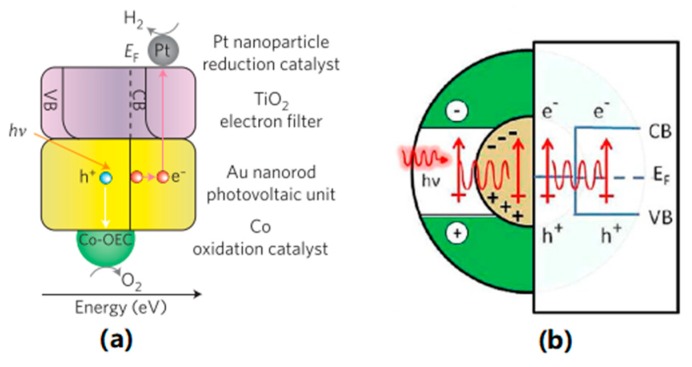
(**a**) Schematic diagram of a hot-electron induced photosynthesis device with Pt reduction catalyst, TiO_2_ electron filter, Au nanorod photovoltaic unit, and Co catalyst. Co-CEC: oxygen evolution cocatalyst [23] (Reprinted from Nature Nanotechnology with permission from Springer Nature, copyright 2013), (**b**) Schematic diagram of PIRET from the LSPR dipole to a semiconductor shell. Reproduced with permission from [21]. Copyright American Chemical Society, 2012.

HEI describes the process in which the plasmonic materials harvest photon energy, which is normally below the band gap of semiconducting material, in the first step, and subsequently inject the excited hot electrons directly into the conduction band of the semiconductor. The excitation of the electrons in plasmonic materials generates energetic electron-hole pairs. When the energy of the generated carries is higher than those from thermal excitations at ambient temperatures, those carries become hot carriers and could be injected into the semiconductor. As the light absorber here is the plasmonic material itself, there is no limitation of the harvested photon energy from the band gap of the semiconductors [22]. Considering that HEI being a two-step energy transferring process with a relatively low efficiency, the distance between plasmonic nanostructure and the acceptor molecules much be small as shown in Figure 4a. Most importantly, HEI offered the unique possibility to harvest the energy below the band gap of semiconducting materials, normally in visible to NIR range which can not be fulfilled by any other mechanisms.

### 3.3. Plasmon Induced Resonance Energy Transfer (PIRET)

PIRET is a process in which the energy from the photons is transferred to the semiconductors indirectly with the help of the plasmonic oscillation, as demonstrated in Figure 4b. This energy transfer process can be accomplished through a dipole-dipole interaction or through the generated electromagnet field of the plasmonic nanostructures. Therefore, the generation of electron-hole pair takes place only in the semiconductors and the spectral frequency of absorbed light with the PIRET mechanism cannot go over the band edge of the corresponding semiconductor. Since no direct contact is required between the plasmonic material and semiconductors. PIRET usually becomes one of the main pathways to plasmonic enhancement when the plasmonic absorption band overlaps with the semiconductor absorption band [21].

### 3.4. Photothermal Effect

When illuminated, plasmonic nanostructures enhance the optical absorption by plasmon resonances. Meanwhile, the free electron gas is heated by electron-electron scattering within 100 fs. The energy transfers from electron gas to metal lattice via electron-phonon scattering over several picoseconds, and finally is delivered to the surrounding medium, e.g. the electrolyte, in 100 ps. Regarding this field, Ahmadivand et al. fabricated a quadrumer nanocluster containing silver core-shell structure mixed with carbon nanospheres. A thermal heat flux of 93.3 μW cm^−2^ was obtained and the temperature was raised by 172 K [24]. Toroghi and Kik investigated and optimized a plasmonic Ag-Au-Ag trimer structure with rapid and localized heat generation, showing a promising potential in photothermal applications [25]. Besides, Zhixing Gan et al. demonstrated that the contribution of the photothermal effect in a graphene-based photocatalytic system could be up to 38%, indicating that the photothermal effect plays an important role in light-matter interaction process [26].

## 4. Enhancement of Photoelectrolysis Using Aluminum Plasmonic Systems

Aluminum is one of the most abundant metallic elements on earth. Asa plasmonic material, aluminum has many advantages over Ag and Au. The plasmonic resonance frequency of aluminum nanostructures is typically located in the UV region, which is not covered by Au and Ag. In addition, by controlling the size and morphology, a broadband plasmonic resonance from UV to NIR can be easily achieved. These properties of aluminum make it a promising candidate in most systems that require plasmonic enhancement. Many people have reported utilizing aluminum plasmonic nanostructures in the fields of optical biosensing [4], light harvesting [27], photocatalysis [28] and photodetection [29].

There are mainly two types of aluminum nanostructures: nanoparticles that primarily exhibit LSPR effects and nanofilms which primarily exhibit SPP effects. Plenty of methods to nanostructure aluminum are available but due to its oxyphilic property, the synthesis of nanostructures is usually performed in a vacuum or protective atmosphere. 

**Figure 5:**
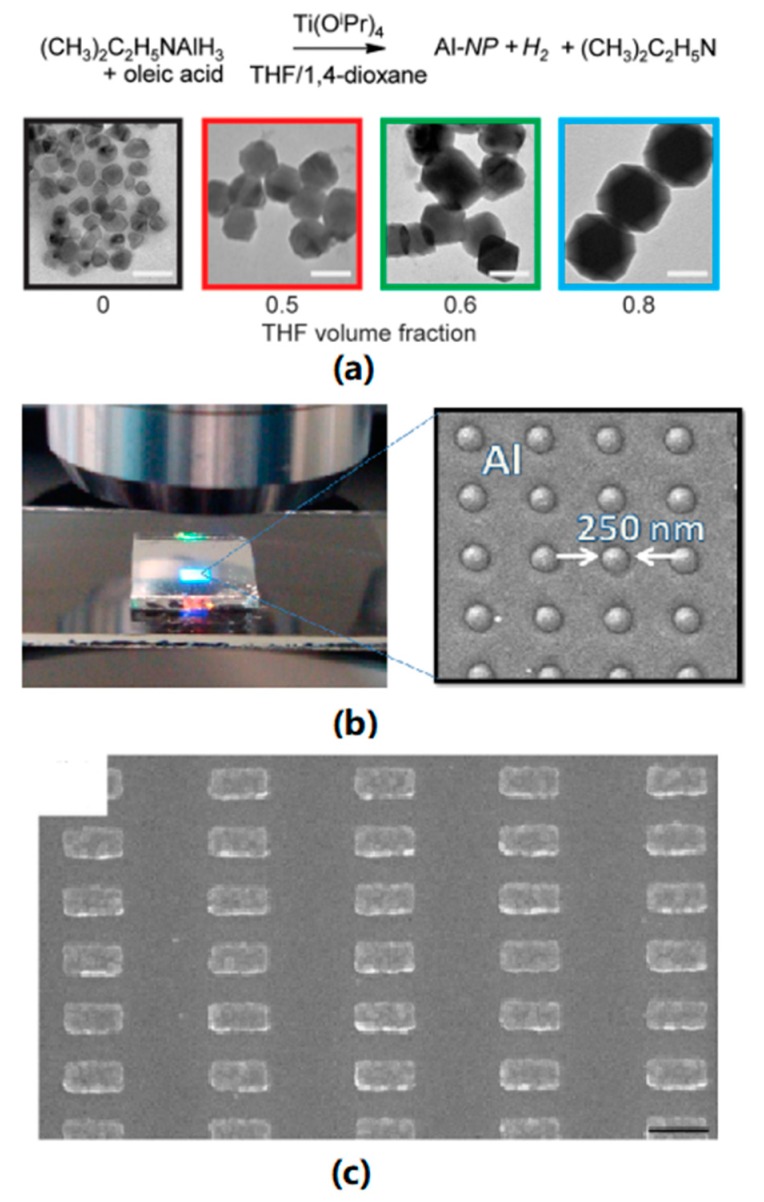
(**a**) Size controllable synthesis method and TEM image of aluminum nanocrystals, scale bar = 100 nm. Reproduced with permission from [30]. Copyright American Chemical Society, 2015. (**b**) SEM image of Al nanodisk arrays with a diameter of 250 nm and a period of 500 nm [4] (Reprinted from Biosensors under CC-BY license), (**c**) SEM image of rectangular aluminum nanorod arrays, scale bar = 200 nm. Reproduced with permission from [31]. Copyright American Chemical Society, 2014.

Chemical reduction is one of the common approaches to obtain aluminum nanoparticles, where to avoid the oxidation of synthesized particles, the presence of oxygen must be strictly limited. McClain et al. reported the synthesis of icosahedral and trigonal bipyramidal aluminum nanocrystals with organic precursor and organic reducing agent, as shown Figure 5a. The synthesized nanocrystals have a controllable size from 70 to 200 nm and can exhibit plasmonic resonance in UV region. S. Lee et al. reported a method to synthesize Al nanoparticles by laser ablation. The size of the product could be tuned by the laser power and centrifugation rate from 20 nm to 50 nm. Mahendiran et al. utilized a sonoelectrochemical method to synthesize aluminum nanoparticles with a much smaller size, from 10 to 20 nm.

Lithography with different templates can produce Al films with various morphologies. Lecarme et al. reported the fabrication of Al nanorod arrays with e-beam lithography as shown in Figure 5c. The 130 × 65 nm rectangular nanorods had a resonance wavelength in the NIR region. Barrios et al. reported Al nanohole arrays with highly controllable hole diameters as demonstrated in Figure 5b. UV-Vis spectra demonstrated that an Al film with an array of 500 nm holes exhibits plasmonic resonance in the visible region. Colloidal lithography is another efficient method to synthesize periodic plasmonic thin films. Schmidt et al. used polystyrene particles as a template to synthesize Al nanoholes with tunable SPP and LSPR wavelength [32]. Typical methods of synthesizing aluminum nanostructures are listed in Table 1.

As mentioned before, incident light with frequencies that match the resonance frequency of a plasmonic material will excite plasmons in either LSPR or SPP mode. For aluminum plasmonic nanostructures, the resonance frequency is usually located in the UV-Vis range. Meanwhile, the most common semiconductors for water splitting, including TiO_2_, α-Fe_2_O_3_, and WO_3_, have their absorption maxima within the same region, hence aluminum plasmonic can boost the photocatalyst or photoelectrochemical reactions when coupled with these materials. 

**Figure 6:**
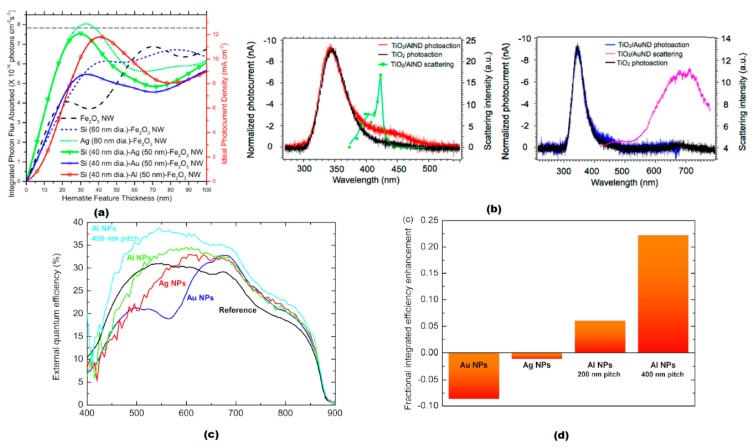
(**a**) Integrated absorbed photon flux and Ideal photocurrent density of Fe_2_O_3_, Si-Fe_2_O_3_ and Si-Metal-Fe_2_O_3_ core-shell nanowires. Reproduced with permission from [37]. Copyright American Chemical Society, 2016. (**b**) Photocurrent and scattering spectrum of TiO_2_ thin films with or without Al or Au nanodimers. Reproduced with permission from [38]. Copyright American Chemical Society, 2016. (**c**) Experimental photocurrent spectra and (**d**) Integrated EQE enhancement provided by different nanoparticle (Au, Ag, Al) arrays [41].

Ramadurgam et al. have demonstrated, using computer simulation, that a Si-Al-Fe_2_O_3_ core-shell nanowire structure could achieve a solar to hydrogen efficiency of up to 14.5% under AM 1.5G light, which is greater than 90% of hematite’s maximum theoretical limit [37]. In this system, the Al metallic core, with a tunable LSPR resonance frequency in the UV-Vis, can strongly enhance the local electromagnetic field and thus increase absorption in the hematite shell. The Si in this system acts as a second semiconductor that can effectively reduce the overpotential of the hematite. Also, as demonstrated in Figure 6a, Si-Al- Fe_2_O_3_ systems have a better photon flux compared to Au induced systems. This work established core-shell nanoparticles with Al plasmonic cores and semiconductor shells as potential structures for improving the efficiency of large-scale water splitting devices.

Zhou et al. utilized aluminum nanodimers underneath a TiO_2_ thin film to enhance, via the surface plasmon, system performance [38]. Al nanodimers have a scattering spectral peak closer to the visible region compared to Au nanodimers as demonstrated in Figure 6b. The Al nanodimer arrays had a plasmonic resonance overlapped with the absorption profile of the TiO_2_ layer, in the spectral range from 370 to 475 nm, and thus an increased photon to current efficiency was observed in this region. Although only a small portion of the thin film was covered by Al nanodimers, the system achieved an enhancement of up to 27.8% in local oxygen evolution rate.

In Kakavelakis et al.’s work, aluminum nanoparticles were applied in an organic photovoltaics system giving an efficiency enhancement of up to 30% [39]. They utilized aluminum nanoparticles from 10 to 70 nm in size and incorporated them within the organic photoactive layer. Both the current density and the incident photon-to-electron conversion efficiency increased drastically for those samples with Al NPs due to the contribution of the enhanced local field from the LSPR mode of Al. Recently, Liang et al. also utilized aluminum nanohole arrays in organic photovoltaics [40]. A self-assembled nanoparticle template was used to fabricate the size-tunable Al nanodisk array on the top of a TiO_2_ film. The average power conversion efficiency could be enhanced significantly by the plasmonic array.

Hylton et al. fabricated an aluminum nanodisk array on a GaAs photodiode to obtain a plasmonic-enhanced solar cell [41]. They demonstrated that the LSPR mode of the metal disk arrays could lead to an increased optical path length over broad bands as well as an enhanced absorption. The Al nanostructures they used possessed a remarkable blue shift and low optical absorption when compared to Au and Ag (Figure 6c), which enabled truly broadband plasmonic enhancement of photocurrent and integrated efficiency. Figure 6d indicates that such enhancement in Al-induced systems is significantly greater than that in Au and Ag based systems. Mahani and Mokhtar employed a graphene-aluminum hybrid structure with a morphology of nanocross arrays for plasmonic enhanced solar cells. The graphene-based substrate replaced ITO and was plasmonically enhanced by the Al nanocrosses on top, resulting in an impressive increase in photocurrent density from 11.82 to 17.05 mA/cm^2^.

L. Zhou et al. demonstrated the application of aluminum nanocrystals as a plasmonic photocatalyst in hydrogen dissociation [42]. Hydrogen dissociation, as an important step in hydrogen evolution reactions (HER), is critical to the efficiency of water splitting devices. In their work, they showed that the plasmonic enhancement is due to injection from the aluminum of hot electrons which form both as a result of surface plasmon decay and photoexcitation of Al’s own interband transitions which weaken the H−H bond. Also, in the Halas group, Chao Zhang et al. utilized a precisely designed Al-Pd nanosk heterodimer as a photocatalyst for the hydrogen dissociation reaction [43]. Hot electrons were generated from plasmonic decay in the Al nanoantenna with Pd as a catalytic reactor. Typical Al-based plasmonic structures for use in energy applications are listed in Table 2.

## 5. Enhancement of Photoelectrolysis Using Copper Plasmonic Systems

Copper is another non-noble and earth-abundant element. Cu nanoparticles exhibit tunable plasmonic resonance frequency ranging from the visible to NIR range. Although the real and imaginary dielectric function of Cu is similar to Au, the former is ca. 6500 times cheaper and could be a promising alternative metal. Furthermore, Cu has the second-highest conductivity among all the metals, only inferior to Ag. Owing to these promising properties, Cu is considered to have great potential in plasmonic enhanced photocatalysis and photoelectrochemical catalysis. In terms of water splitting, Cu-based nanomaterials have been developed for hydrogen evolution reactions (HER) and oxygen evolution reactions (OER), though bare Cu nanoparticles (CuNPs) could not be directly used in OER due to oxidation of copper. General approaches to the synthesis of Cu plasmonic nanocrystals include chemical reaction, photoreduction, and solvothermal synthesis. Chemical methods include wet-chemical and microwave (MW)-assisted approaches. Other methods such as laser irradiation have also been reported [46].

Work devoted to the synthesis of Cu nanoparticles is summarized in Table 3. A thorough summary of previous CuNP synthesis achievement is beyond the scope of this work, please refer to other reviews [47,48,49]. The most commonly adopted wet-chemical approach involves the process in which copper salts are reduced by reducing agents. Reducing agents used include hydrazinium hydroxide, hydrazine sodium borohydrate, L-Ascorbic acid and even plant extract. To illustrate, Liu and his coworkers selected hydrazine for the reduction of CuS with poly(vinylpyrrolidone) (PVP) and cetyltrimethylammonium bromide (CTAB) mixed capping. Kaur et al. reported 50–54 nm CuNPs obtained from copper-surfactant complex reduced by sodium borohydrate. Generally, the diameter of CuNPs reported ranges from 2 to 200 nm and displays LSPR effects. Precise size control of CuNPs has been realized from a micelle-based synthesis by the Wu group, with mean diameters ranging from 5 to 15 nm with a standard deviation of 1.4 nm [50]. Liu’s group reported another fabrication technique, where CuNPs were obtained by photoreduction of Cu(OAc)_2_ under vacuum to prevent oxidation [51]. The CuNPs exhibited a peak at 580 nm from SPR.

Other than nanoparticles, the use of nanowires, nanocubes, and nanoplates have been reported. Jin’s group successfully synthesized both Cu nanocubes and pentagonal/tadpole-like Cu nanowire with glucose as the reductant and hexadecylamine as capping source [57]. Pastoriza-Santos et al. prepared Cu nanoplates with hydrazine as the reducing agent and PVP as stabilizer [58].

**Figure 7:**
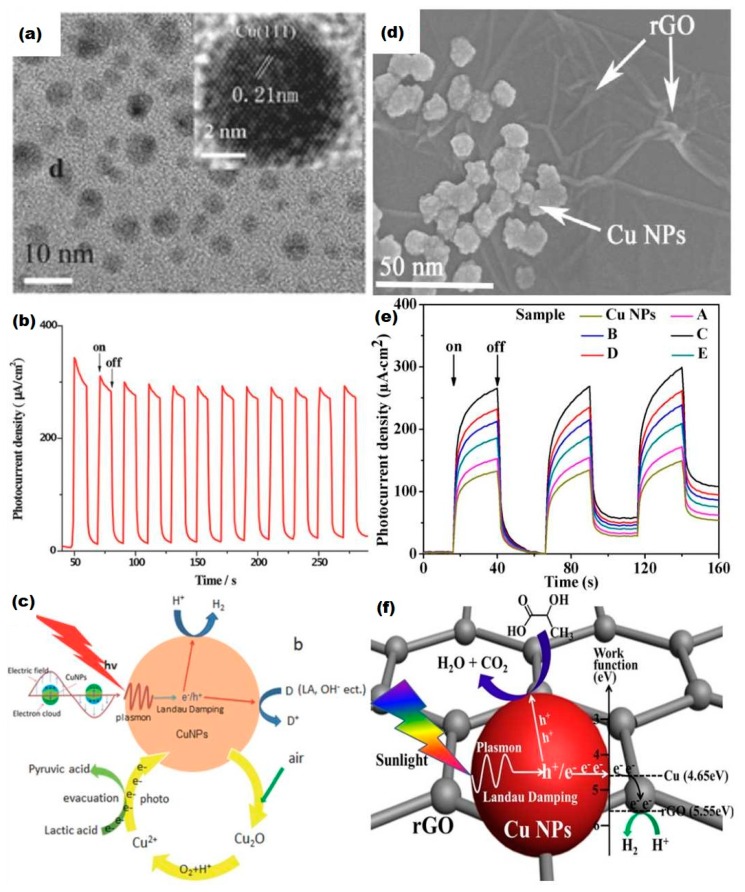
Plasmonic Cu and Cu-based hybrid materials and their photocatalytic application (**a**) HRTEM images of CuNPs synthesized by photoreduction, (**b**) Transient photocurrent responses of the CuNPs under UV–vis light irradiation, (**c**) Schematic of CuNP photocatalysis. Reproduced with permission from [51]. Copyright John Wiley and Sons, 2015. (**d**) SEM image of CuNP/rGO, (**e**) Transient photocurrent responses of CuNP/rGO, (**f**) Schematic of HER of CuNP/rGO. Reproduced with permission from [59]. Copyright Elsevier, 2018.

According to the intrinsic optical property of Cu, the performance of single Cu nanocrystal (NC) catalyst could be no better than rationally designed heterojunction semiconductors in photoelectrolysis. Nevertheless, Liu et al. employed CuNPs as photoelectrochemical catalysts individually. PEC measurements were carried out and a moderate H_2_ evolution rate of 35 mmol g^−1^ h^−1^ was achieved [51]. In Figure 7a, the TEM image displays CuNPs with a small diameter of ~10 nm. A stable photocurrent response is confirmed in Figure 7b. Figure 7c briefly describes the mechanism of CuNPs enabled photocatalysis. CuNPs firstly receive energy from illumination and form resonant collective oscillations, then plasmon decay occurs and generates an electron-hole pair through non-radiative Landau damping [51]. Afterward, electrons react with protons to form hydrogen.

On the other hand, more attention has been devoted to Cu-based hybrid nanostructures as the plasmonic enhancer for the HER. ZnO [60], TiO_2_ [61], graphene and its derivatives [59] have been used in cooperation with copper to form photonic enhanced catalysts. For semiconductors like ZnO or TiO_2_, Cu could expand the light response region from UV to the visible region and promote the charge carrier transportation and separation. To illustrate, Li et al. decorated ZnO thin film with CuNPs via a two-step dc magnetron sputtering approach [60]. The density of CuNPs could be readily tailored by the sputtering parameters. The as-prepared Cu/ZnO thin films were used as photoanodes for PEC water splitting and a six-fold increase in photocurrent density, compared to pure ZnO films, was obtained under visible illumination [60]. The outstanding enhancement in photocurrent could be attributed to both light trapping and hot electron injection from SPR. 

Zhang et al. successfully decorated plasmonic CuNPs onto reduced graphene oxide (rGO) (Figure 7d) and this nanohybrid photocatalyst reached a H_2_ evolution rate of 59 mmol g^−1^ h^−1^ under solar light irradiation [59]. In Figure 7e, the optimized sample C contains 1.0 wt% of rGO nanosheets. As shown in Figure 7f, plasmonic oscillation occurs in CuNPs, then decay to electron-hole pairs. It is proposed that electrons spontaneously transfer to rGO and reduce protons yielding hydrogen.

Prior to Zhang’s work, Lv et al. developed a Cu-graphene cocatalyst immobilized on TiO_2_ [52]. The introduction of graphene improved electron transport and suppressed charge recombination. A synergetic effect between copper and graphene brought a promising hydrogen efficiency comparable to Pt. However, a transition process from Cu to CuO and Cu_2_O occurred under UV-visible light irradiation. In other words, Cu was gradually oxidized during the photocatalytic hydrogen evolution reaction. The instability of Cu nanoparticles remains a significant challenge and work to negate this problem will be discussed below. 

To date, Cu plasmonic nanostructures for water oxidation are much less popular than those formed from Au and Ag. The bottleneck is the increasing complexity in building chemically stable Cu nanostructures with intense plasmon resonances [58]. Herein, the poor chemical stability of Cu in ambient condition becomes the primary obstacle. Cu nanoparticle oxidation would occur rapidly in air and bring an optical property that fluctuates with time. It is commonly known that nanomaterials have a large surface-to-volume ratio and more surface atoms and dangling chemical bonds, thereby having even higher reactivity than their bulk state [62]. The high reactivity and instability become a common problem for nanomaterials, which hinder them from wider applications. With regard to Cu plasmonics, several strategies have been adopted to prevent Copper oxidation, such as providing anoxic environments, building core-shell structures based on Cu core covered with adsorbates (thiols [63], polymer [58,61]), chemical corrosion inhibitors (silica [64], BTAH [65]) or alloys [66]). 

Example of polymer stabilized Cu composite electrodes are PVP@CuNP-TiO_2_ and PVA@CuNP-TiO_2_ prepared by Yamaguchi et al. The copper was stabilized by PVP/PVA, but the incident photon to current conversion efficiency (IPCE) was extremely low at 0.01% [61]. The organic coating layer can contribute to the removal of the oxidized donor to the electrolyte and the supply of electron donor from the electrolyte to the CuNPs. Yet, PVA had no light response and poor permeability to electrolyte ions. It appears that the organic coating improves the chemical stability at the expense of light conversion efficiency.

Several ligands were studied by Kanninen’s group on their effect on oxidation stability of CuNPs [63]. Thiols were confirmed to improve oxidation resistance and the effectiveness increased with longer chain length. Oleic acid presented better results at a high ligand-to-Cu ratio. Unfortunately, these ligand-coated CuNPs were not employed as a plasmonic enhancer for photocatalyst, though some of them exhibit stable plasmonic response.

Susman’s group selected benzotriazole (BTAH) as the corrosion inhibitor [67]. Glass substrates were coated with CuNPs via wet chemical deposition, and further stabilized by BATH, which was effective at neutral to alkaline pH. The structure of the BATH layer was not unconfirmed. It was claimed that either a Cu(I)-BATH multilayer complex or a monolayer of BTAH was formed on top of the CuNPs surface [67]. The extinction efficiency of the as-fabricated CuNP composite films was comparable to gold. Tanaka et al. alloyed Cu with Au and a strong and stable absorption peak at λ = 620 nm arose from surface plasmon resonance of Cu-Au NPs [66]. Silica-coated CuNPs were fabricated from the reaction between Cu nanoparticles and sodium silicate solution [64]. The as-prepared Silica@Cu showed fantastic chemical stability in air. However, these post-coated CuNP compounds were not employed in PEC testing. DeSario et al. incorporated CuNPs into a TiO_2_ aerogel nanoarchitecture in which oxygen vacancies could readily occur, and extensive interfacial contact between Cu and TiO_2_ was established [68]. TiO_2_ acted as a reducing metal oxide support. The plasmonic properties of CuNPs were stable during UV-visible light driven photoelectrochemical measurement and the generation of electron-hole pairs from Cu SPR was confirmed.

Although several corrosion inhibition methods have been developed and applied to stabilize Cu nanoparticles, very limited success has been achieved for Cu to act as a stable plasmonic enhancer in photoelectrolysis. CuNPs normally act as a plasmonic material contribute to enhanced light harvesting and promote charge transfer/separation for semiconductors. The three plasmonic enhancement process (resonant energy transfer (RET), direct electron transfer (DET) and local electromagnetic field enhancement (LEMF)) are very sensitive to the morphology and shape of metal plasmonic particles. For copper, various SPR peaks ranging from visible to NIR region could be obtained by tuning the diameter of Cu nanospheres or the aspect ratio of Cu nanorods. Furthermore, the incident light onto CuNPs should have energy higher than the bandgap of Cu-decorated semiconductor for effective LEMF. Substantial spectral overlap of the semiconductor and Cu is required for PIRET, while intimate contact could ensure the occurrence of hot electron injection/DET. DET and LEMF usually entangled and would not work with a rather thick insulating space layer outside CuNPs, which was coated for stabilization. In terms of this problem, the possible solutions could be: i) Building ultrathin insulating layer like silica, which may not dampen plasmonic enhancement. ii) Alloying Cu with another metal. Ranno et al. did computational design and predicted Cu with Na or K could have perfect DET performance for water splitting, but Na and K are even more reactive than Cu [69]. Ag and Au are good candidates as well, though much more costly. Nevertheless, it is still worth trying mixing Cu with Zn, Al and other earth-abundant and stable metals. iii) Cu could be encapsulated by metal-organic framework (MOF), the latter could both provide active sites and prevent oxidation of copper. 

**Figure 8:**
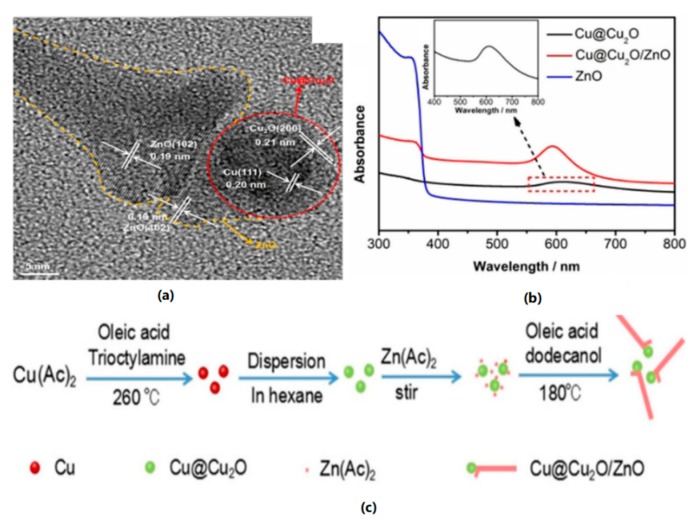
(**a**) HRTEM image of Cu@Cu_2_O/ZnO nanohybrid, (**b**) UV-Vis spectra of Cu@Cu_2_O, Cu@Cu_2_O/ZnO nanocomposites, and ZnO nanorods, (**c**) Schematic of the fabrication process of Cu@Cu_2_O/ZnO heterojunction nanocomposite. Reproduced with permission from [62]. Copyright John Wiley and Sons, 2018.

It is noteworthy that copper oxide or hydroxide can be employed as the protecting layer over the copper surface. Du et al. deposited CuO and Cu(OH)_2_ on a Cu substrate. Then the Copper worked as a transparent and robust electrocatalyst for water splitting and current densities of 10 mA cm^−2^ were obtained at overpotentials of 580 mV [67]. As shown in Figure 8c, Lou et al. adopted a hot-injection synthesis approach to preparing lollipop-shaped Cu@Cu_2_O/ZnO heterojunction nanostructure, where Copper plasmonic particles were embedded in CuO shell linked with ZnO nanorods (Figure 8a). ZnO improved the stability of the Cu@Cu_2_O while the latter assisted in the absorption of light (Figure 8b), and drastically promoted the hydrogen evolution reaction at the original ZnO nanorods [62]. The Cu@Cu_2_O/ZnO heterojunction offered efficient electron transfer through Cu→Cu_2_O→ZnO route and retarded electron-hole recombination. The LSPR-mediated hot electron injection was demonstrated to contribute to the elevated photocatalytic performance. These copper oxide layers inhibited corrosion of copper and facilitate sustainable water splitting without electrode degradation. Hence, it is possible to allow partly Cu oxidation with controllable plasmonic resonance properties. 

Typical photocatalysis or photoelectrochemical catalysis applications of Cu-based hybrid nanomaterials are listed in Table 4. Apart from water splitting, Cu and Cu-based hybrid nanomaterials have much wider applications such as catalysis in organic transformations [74], photocatalysis in CO_2_ reduction [75], electrocatalysis in fuel-cell-related reactions [76], and light-induced antibacterial activity [77]. For instance, CuNP-TiO_2_ nanoflower composite films were manufactured by a hydrothermal synthesis followed by microwave-assisted reduction. This composite displays visible light harvesting based on the LSPR effect of CuNPs: excitation of electron-hole pairs in TiO_2_ was boosted by the enhanced near-field and the charge recombination was retarded. The efficiency of reduction of CO_2_ to methanol therefore exhibited a six-fold enhancement to 1.8 μmol cm^−2^ h^−1^ under UV and visible light irradiation comparing to pure TiO_2_ film [75]. Kayano et al. made copper-deposited TiO_2_ thin films and obtained effective bactericidal performance [77]. The cell envelope decomposition firstly occurred promoted by a photocatalytic process, then the cytoplasmic membrane was disrupted with the permeation of Cu ions.

## 6. Conclusions

Plasmonic enhanced photoelectrolysis has been well studied in various systems with the combination of metal oxide semiconductors and noble plasmonic metals while less work has been done with aluminum and copper based photoelectrolysis systems. Reported modeling and experiment results have revealed the outstanding potential of non-noble plasmonic materials and the potential synergistic effect of a composite of those non-noble materials. Being easy to fabricate, abundant in the earth and low cost, aluminum and copper based plasmonic nanostructures have the potential in large-scale applications in industry. Moreover, the existing results have shown us the superiority of these non-noble metals in multi-pathways and broadband plasmonic enhancement through electric field enhancement, PRIET, HEI, and photothermal effect. Hence, aluminum/copper plasmonic nanostructures and their alloys can act as a novel enhancement strategy for almost all photoelectrolysis devices.

Considering all noble and non-noble plasmonic materials, not a single plasmonic nanostructure has been found to cover the whole solar spectrum from UV to NIR. In view of the plasmonic resonance range of aluminum and copper, the plasmonic response of Al covers from UV to visible range, while the plasmonic response of Cu covers from visible to NIR range, it is possible to design a photoelectrolysis system which is capable of achieving a broadband plasmonic enhancement completely overlapping with the solar spectrum. Although it remains highly challenging to fabricate a photoelectrolysis device due to the chemical instability of Al and Cu, the study of aluminum and copper based plasmonic enhanced photoelectrolysis could fundamentally improve the insight of both non-noble metal plasmonic nanostructures and plasmonic induced light-matter interaction in photoelectrolysis process, paving the way for a reliable, low-cost and large-scale light harvesting technology.

## Figures and Tables

**Table 1:** Typical synthesis and fabrication methods of Al plasmonic nanostructures.

Method	Precursor	Morphology	Size	References
Chemical reduction	(CH_3_)_2_C_2_H_5_NAlH_3_	Nanocrystal	70–200 nm in diameter	[30]
E-beam lithography	Al	Nanorod arrays1	130 × 65 nm	[31]
E-beam lithography	Al	Nanohole arrays	200–800 nm in diameter	[4]
Colloidal lithography	Al	Nanoholes	67 nm in diameter	[32]
Sonoelectrochemical	AlCl_3_, LiAlH_4_	Nanoparticles	10–20 nm in diameter	[33]
Laser ablation	Al	Nanoparticles	20–50 nm in diameter	[34]
Anodic oxidation	Al	Porous Al	Porous film	[35]
Deposition/Dewetting	Al	Al/Al_2_O_3_ nanoparticle arrays	12–25 nm	[36]

**Table 2:** Typical energy applications of aluminum as a plasmonic material.

Application	Structure	Performance	Reference
Photocatalyst	Si-Al-Fe_2_O_3_ core-shell nanowires	(theoretical) 14.5% solar to hydrogen efficiency	[44]
Photoelectrochemical	Al nanodimer and TiO_2_ thin film	27.8% increase in the local oxygen evolution rate	[38]
Organic photovoltaics	Aluminum nanoparticles	30% efficiency enhancement	[39]
Organic photovoltaics	Aluminum nanodisk array	7.28% to 8.04% enhancement in PCE	[40]
Solar cells	Aluminum nanodisk array	38% external quantum efficiency at 530 nm	[41]
Photocatalyst	Aluminum nanocrystals and GaAs	1.5e5 c/s HD Rate under 4 kW/cm^2^ laser illumination	[42]
Photocatalyst	Al-Pd nanodisk heterodimers	28 nmol/s HD Rate at 450 nm	[43]
Organic solar cells	Graphene-aluminum nanocross arrays	11.82 to 17.05 mA/cm^2^ photocurrent density	[45]

**Table 3:** Typical synthesis of copper nanoparticles.

Method	Precursor	Reducing Agent	Stabilizer	Size	Product	References
Wet-chemical	Cupric chloride	Hydrazinium hydroxide	CTAB	5–15 nm	CuNPs	[50]
Photoreduction	Cu(OAc)_2_	Irradiated with a xenon lamp	-	10 nm	CuNPs	[51]
Wet-chemical & MW-assisted	Cupric nitrate	Terminalia arjuna bark extract	Terminalia arjuna bark extract	23 nm	Cu-MWCNTs	[52]
Wet-chemical	Copper-surfactant complex	Hydrazine hydrate;	Deprotonated polyacrylic acid	40–85 nm	CuNPs	[53]
Wet-chemical	Copper-surfactant complex	Sodium borohydrate	Deprotonated polyacrylic acid	50–54 nm	CuNPs	[53]
MW-assisted	copper acetate	Sodium hydroxide	-	7 nm	CuNPs	[54]
MW-assisted	copper nitrate	L-Ascorbic acid	-	9 nm	CuNPs	[54]
Micelle method	copper(II) sulfate pentahydrate	Sodium borohydrate	sodium dodecyl sulfate	~2 nm	Cu nanoclusters	[55]
Ionic-liquid (IL)-assisted synthesis	Microsized copper particles (1–5 µm)	1-butyl-3-methylimidazolium tetrafluoroborate	-	20–200 nm	CuNPs	[56]

**Table 4:** Performance of Cu-based hybrid nanomaterials as photocatalysts (PC) or photoelectrochemical electrodes (PEC).

Structure	Reaction	Performance	References
CuNP	PC-HER	Hydrogen evolution rate of 35 mmol g^−1^ h^−1^	[51]
CuNP-ZnO composite	PEC-OER	0.017 mA cm^−2^ at 0.5 V (vs. SCE) under visible light illumination	[60]
CuNP/rGO	PEC-HER	Hydrogen evolution rate of ~59 mmol/h/g	[59]
CuNP@Cu_2_O/ZnO	PC-HER	Hydrogen evolution rate of ~1.47 mmol/h/g	[62]
CuO/Cu(OH)_2_@Cu substrate film	PEC-OER	10 mA cm^−2^ at an overpotential of 580 mV	[67]
Nanorods with Cu(OH)_2_-CuO@Cu core-shell structure	PEC-OER	10 mA cm^−2^ at an overpotential of 417 mV	[70]
CuO-modifed TiO_2_	PC-HER	Hydrogen evolution rate of 64.2–71.6 mmol/h/g	[71]
CuO-TiO_2_	PC-HER	Apparent quantum yield of 5.1%	[72]
Cu(OH)_2_-nanocluster-modified TiO_2_	PC-HER	Hydrogen evolution rate of ~3.4 mmol/h/g, quantum efficiency (QE) ~13.9%	[73]
